# Gerontologizing the Learning Health System – The Electronic Frailty Index as a Passive Digital Marker for Frailty

**DOI:** 10.1093/geroni/igaf122.1279

**Published:** 2025-12-31

**Authors:** Kathryn Callahan

**Affiliations:** Wake Forest University School of Medicine, Winston-Salem, North Carolina, United States

## Abstract

Whether defined as a phenotype (Fried et al.) or through a frailty index (Rockwood et al., age-associated deficit accumulation), frailty metrics capture a unique description of an individual’s, or population’s, health in aging. Frailty has been slow to implement in healthcare systems, likely due to burdensome assessments. Building upon the Rockwood frailty index and the Clegg et al. electronic health record (EHR) based frailty index (eFI) in England, our team developed and implemented an eFI within the Wake Forest University School of Medicine (WFUSM) EHR, derived from personalized data collected during routine clinical care. Consistent with established methods, we compiled 54 age-associated deficits. eFI scores are reported as a simple proportion, with cut-points denoted as “Fit” (eFI≤0.10), “Pre-Frail” (0.100.21). Across 22+ published articles in multiple healthcare domains, eFI scores are consistently associated with adverse health outcomes for older adults. In primary care, the c-statistics for frailty include (adjusted for age, sex, race/ethnicity, past utilization) 0.79 for mortality, 0.74 for acute utilization, 0.79 for emergency department visits, 0.74 for injurious falls. Post-operative frail (eFI>0.21) older adults experienced an elevated hazard of six-month mortality (HR 2.86, 1.84-4.44), and elevated odds of 30-day readmissions (OR 2.46, 1.72-3.52) and post-acute services (OR 1.68, 1.36-2.08). Frail older adults with solid tumors undergoing chemotherapy experienced greater hazard of hospitalization (HR 3.23, 2.23-4.69) and lower mean overall survival (19m in frail vs 54m in fit). We are testing care pathways to improve quality of life and health outcomes in frailty.

